# Initial WNT/β-Catenin Activation Enhanced Mesoderm Commitment, Extracellular Matrix Expression, Cell Aggregation and Cartilage Tissue Yield From Induced Pluripotent Stem Cells

**DOI:** 10.3389/fcell.2020.581331

**Published:** 2020-10-30

**Authors:** Ursula Kreuser, Justyna Buchert, Alexandra Haase, Wiltrud Richter, Solvig Diederichs

**Affiliations:** ^1^Research Center for Experimental Orthopaedics, Heidelberg University Hospital, Heidelberg, Germany; ^2^Leibniz Research Laboratories for Biotechnology and Artificial Organs (LEBAO), Department of Cardiac, Thoracic, Transplantation, and Vascular Surgery, Hannover, Germany

**Keywords:** induced pluripotent stem cells, cartilage, WNT/β-catenin, extracellular matrix, cell survival, aggregation

## Abstract

Mesodermal differentiation of induced pluripotent stem cells (iPSCs) *in vitro* and subsequent specification into mesodermal derivatives like chondrocytes is currently afflicted with a substantial cell loss that severely limits tissue yield. More knowledge on the key players regulating mesodermal differentiation of iPSCs is currently needed to drive all cells into the desired lineage and to overcome the current need for intermediate cell selection steps to remove misdifferentiated cells. Using two independent human iPSC lines, we here report that a short initial WNT/β-catenin pulse induced by the small molecule CHIR99021 (24 h) enhanced expression of mesodermal markers (PDGFRα, *HAND1, KDR*, and *GATA4*), supported the exit from pluripotency (decreased *OCT4, SOX2*, and *LIN28A*) and inhibited ectodermal misdifferentiation (reduced *PAX6, TUBB3*, and *NES*). Importantly, the initial CHIR pulse increased cell proliferation until day 14 (five-fold), adjusted expression of adhesion-related genes (*CDH3* up, *CDH6* down) and increased extracellular matrix (ECM)-related gene expression (*COL6, COL1, COL3, COL5, DCN, NPNT, LUM, MGP, MATN2, and VTN*), thus yielding more matrix-interacting progenitors with a high aggregation capability. Enhanced contribution to chondrogenic pellet formation increased the cell yield after eight weeks 200-fold compared to controls. The collagen type II and proteoglycan-positive area was enlarged in the CHIR group, indicating an increased number of cartilage-forming cells. Conclusively, short initial WNT activation improved mesoderm commitment and our data demonstrated for the first time to our knowledge that, acting via stimulation of cell proliferation, ECM expression and cell aggregation, WNT pulsing is a key step to make cell selection steps before chondrogenesis obsolete. This advanced understanding of the WNT/β-catenin function is a major step toward robust and efficient generation of high-quality mesodermal progenitors from human iPSCs and toward rescuing low tissue yield during subsequent *in vitro* chondrogenesis, which is highly desired for clinical cartilage regeneration, disease modeling and drug screening.

## Introduction

Induced pluripotent stem cells (iPSCs) have attracted enormous attention in all fields of research because of their immense expandability and their intrinsic ability to give rise to any adult tissue cells, including articular chondrocytes (Diederichs et al., 2019), osteocytes (de Peppo et al., 2013), cardiomyocytes (Lian et al., 2012), and skeletal muscle cells (Shelton et al., 2014). Thus, iPSCs promise to overcome the extremely limited supply of human cells for regenerative medicine, *in vitro* modeling of genetic diseases, and for pharmaceutical screens.

However, *in vitro* differentiation of pluripotent cells into the desired mature phenotype remains challenging. Common strategies for iPSC differentiation *in vitro* aim to recapitulate sequential developmental events in the embryo (Loh et al., 2016). Generation of mesodermal derivatives including cartilage, bone, skeletal muscle or cardiac tissue from iPSCs is, thus, initiated by mesoderm induction. However, the current mesoderm induction protocols are apparently not sufficiently stringent and fail to drive the entire iPSC population into the desired mesodermal phenotype. Consequently, cell selection procedures were applied in many studies to obtain a mesodermal cell population that was sufficiently pure to allow subsequent specification into the desired downstream phenotype like chondrocytes (Umeda et al., 2012; Wu et al., 2013; Dicks et al., 2020), cardiomyocytes (Nguyen et al., 2014; Kadari et al., 2015) or skeletal muscle cells (Mizuno et al., 2010; Kim et al., 2017).

Of note, organogenesis of cartilage and bone as well as skeletal muscles in the embryonic limb bud is initiated by a cell condensation phase, the so-called precartilage or premyogenic condensation (Gould et al., 1972). In line, enrichment of aggregating cells that can condensate was beneficial for chondrocyte derivation from iPSCs *in vitro* not only in our hands (Yamashita et al., 2015; Diederichs et al., 2019), since non-aggregating mesodermal progenitors could not contribute to the forming cartilage (Buchert et al., 2019). Also, for *in vitro* cardiomyocyte differentiation from embryonic stem cells (ESCs) and iPSCs, the initiation of cell condensation appeared highly important and enrichment of aggregating cells in so-called cardiospheres improved subsequent cardiomyocyte homogeneity (Nguyen et al., 2014; Ma et al., 2015). Thus, the capacity to aggregate and condense is a common capability of various mesodermal progenitors. We here hypothesized that establishment of a high aggregation capacity is a functional criterium for the success of mesodermal differentiation and is important for the subsequent development into chondroprogenitors or cardioprogenitors.

However, cell selection and removal of non-aggregating misdifferentiated cells can severely compromise cell and tissue yield, since only a minority of the initial cells remains in the tissue end product. During cartilage generation from human iPSCs, for example, approximately 97% of the starting population did not contribute to the aggregating pellet and was removed and lost in our previous studies (Buchert et al., 2019; Diederichs et al., 2019). This makes iPSC differentiation *in vitro* overly laborious, expensive and inefficient. Thus, stringent induction of more readily aggregating mesodermal progenitors would be highly preferable to allow downstream differentiation into chondrocytes and cardiomyocytes even without prior cell selection.

A strong body of developmental studies in mouse, chick, and zebrafish demonstrated that Wnt/β-catenin signaling is essential in the early embryo for primitive streak induction. This is the initial step of gastrulation, during which specific cells change their phenotype in an epithelial to mesodermal transition, become motile, and ingress to form the mesoderm of the embryo. WNT/β-catenin signaling is activated by canonical WNT ligands and initiates a molecular cascade downstream of the receptor that eventually inhibits the glycogen synthase kinase 3-β (GSK3β). Thereby, the transcriptional co-activator β-catenin is relieved from being marked for proteasomal degradation and translocates into the nucleus where it regulates target gene transcription (Niehrs, 2012). Multiple mouse models with disrupted Wnt/β-catenin signaling failed at correct downregulation of pluripotency markers, induction of gastrulation and establishment of the mesoderm (Haegel et al., 1995; Liu et al., 1999; Yamaguchi et al., 1999; Hsieh et al., 2003; Kelly et al., 2004; Fu et al., 2009; Biechele et al., 2011). After gastrulation, ectodermal Wnt signals were shown to promote proliferation of the neighboring mesoderm cells (ten Berge et al., 2008). Also *in vitro*, Wnt/β-catenin signaling was essential for the induction of mesoderm formation from pluripotent stem cells, according to knockdown and inhibitor studies with mouse ESCs (Lindsley et al., 2006; Naito et al., 2006; Biechele et al., 2011). In turn, WNT/β-catenin signaling enhanced the expression of primitive streak and early mesoderm markers, such as vascular endothelial growth factor receptor 2 (VEGFR2, also called KDR) and platelet-derived growth factor receptor alpha (PDGFRα) in human and mouse ESCs and iPSCs *in vitro* (Ueno et al., 2007; Wang et al., 2010; Umeda et al., 2012; Tan et al., 2013; Shelton et al., 2014; Funa et al., 2015).

Interestingly, Wnt/β-catenin activation was a breakthrough approach for the generation of skeletal muscle cells and cardiomyocytes from iPSCs *in vitro*. An initial 24 or 48 h pulse with the GSK3β inhibitor CHIR99021 (CHIR) enabled subsequent specification of human ESC-derived mesodermal cells into myocytes and myotubes by consecutive growth factor treatments (Shelton et al., 2014). Only CHIR-treated cells, but not controls, upregulated the myoblast commitment transcription factors *MYF5* and *MYOD1* under otherwise identical conditions at days 40 and 50. Secondly, for generating cardiomyocytes, an initial WNT activation pulse and subsequent WNT suppression for 48 h proved essential and tremendously increased the cell yield from less than 1% to up to 98% (Lian et al., 2012; Tan et al., 2013; Kadari et al., 2015; Rao et al., 2016).

We here investigated whether a WNT activation pulse can enhance the development of aggregating mesodermal progenitors from human iPSCs and is, thus, valuable to enhance the currently limited cartilage tissue yield from iPSCs. The aim of this study was, therefore, to unravel the effects of an initial WNT/β-catenin pulse on the *in vitro* differentiation of human iPSCs into mesodermal progenitors and subsequent specification into chondrocytes. Specifically, we asked whether a short initial CHIR pulse would be sufficient to favor mesoderm induction over ectoderm and endoderm formation and whether the development of aggregating mesodermal progenitors with the capacity to condense would be enhanced. Furthermore, we aimed at illuminating the mechanisms underlying the priming of iPSCs with a short initial WNT pulse at initiation of mesoderm formation. We here report for the first time that WNT-induced mesoderm commitment of human iPSCs strongly enhanced the expression of collagens and other ECM-related molecules in mesodermal progenitors and accordingly adjusted cell adhesion molecule-related gene expression. In consequence, the WNT-primed mesodermal progenitors showed a strongly increased capability to aggregate and to undergo mesodermal condensation. Importantly, removal of non-aggregating cells became obsolete and WNT-priming overcame the need for selection of specific subsets of mesodermal cells. Moreover, the WNT pulse was strongly beneficial for subsequent chondrogenesis, since now the majority of cells participated in pellet formation and consequently, the overall tissue yield increased drastically. These revelations can strongly advance chondrogenic hiPSC differentiation in the near future and bring these cells closer to application in regenerative medicine, disease modeling and drug screening.

## Materials and Methods

### IPSC Expansion Culture

The iPSC (IMR90)-4 line (WiCell, Madison, WI, United States), generated from foreskin fibroblasts of a healthy donor (Yu et al., 2007), was routinely cultured on Matrigel^®^ hESC-qualified matrix (Corning Life Sciences, Berlin, Germany) with mTeSR^TM^1 medium (Stemcell Technologies, Cologne, Germany). The medium was exchanged daily. Upon confluency, cells were detached using 1 U/mL dispase (Stemcell Technologies) and reseeded at a 1:5 to 1:10 ratio. The CBiPSC2 line, generated from cord blood-derived endothelial cells from a healthy new-born donor (Haase et al., 2009), was maintained on mouse-embryonic fibroblasts (Thomson et al., 1998), which were proliferation-inactivated by treatment with mitomycin C (Sigma-Aldrich, Taufkirchen, Germany). Expansion medium for the CB line was KnockOut^TM^ Dulbecco’s Modified Eagle’s Medium (DMEM)/F-12 supplemented with 20% KnockOut^TM^ serum replacement, 1% non-essential amino acids (NEAA), 1 mM L-glutamine, 0.2% β-mercaptoethanol (all from Gibco, Invitrogen Life Technologies, Karlsruhe, Germany) and 20 ng/mL fibroblast growth factor-2 (FGF-2, Active Bioscience, Hamburg, Germany). Cells were passaged manually and the medium was supplemented with 10 μM ROCK inhibitor Y27632 (Stemcell Technologies) for re-seeding. Before differentiation, cells were expanded for four passages under feeder-free conditions on Matrigel with mTeSR^TM^1 medium as described above.

### IPSC Differentiation

**FIGURE 1:**
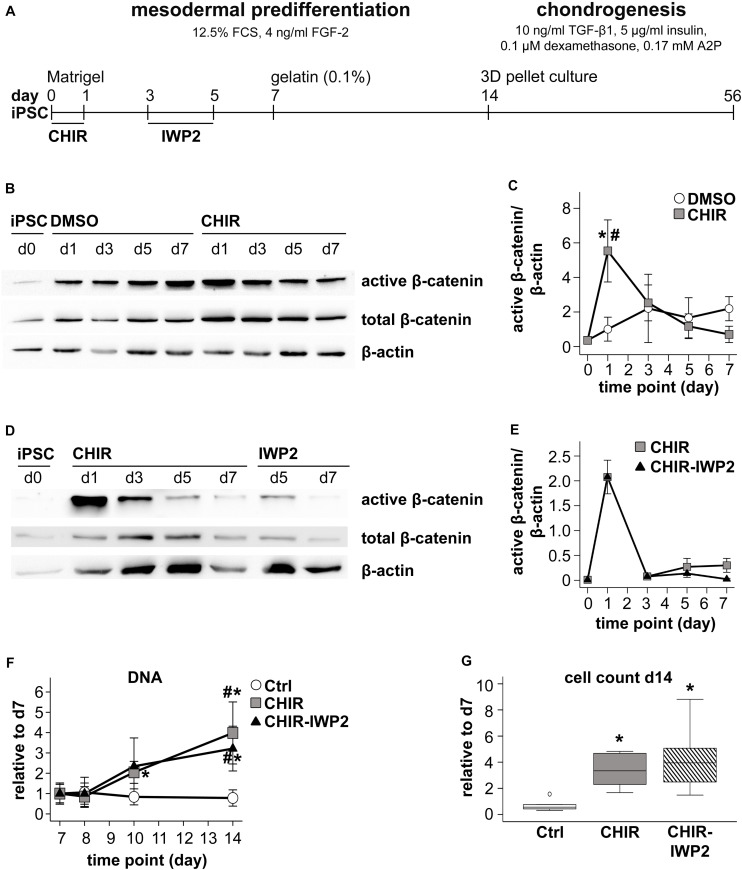
Effects of short initial CHIR treatment on β-catenin regulation and proliferation during IMR-iPSC mesodermal predifferentiation. **(A)** Schematic representation of the iPSC differentiation protocol. Subconfluent iPSC cultures were induced into mesoderm differentiation by switching to serum-containing medium. During the first 24 h cells were additionally treated with 5 μM CHIR99021 (CHIR). Controls received either 0.025% DMSO or remained untreated (Ctrl). After CHIR treatment, the CHIR-IWP2 group received additionally 5 μM IWP2 for 48 h from day 3 to day 5. **(B)** Western blot analysis for active and total β-catenin during the first 7 days of mesoderm differentiation using β-actin as loading control. One representative blot of three is shown. **(C)** Densitometric analysis of Western blots in **(A)** (*n* = 3–5, mean ± SEM, *p < 0.05 vs. Ctrl, paired *t*-test + Bonferroni; ^#^*p* < 0.05 vs. day 0, ANOVA + Bonferroni). **(D)** Western blot analysis of the CHIR and the CHIR-IWP2 group. One representative blot of three is shown. **(E)** Densitometric analysis of Western blots described in **(D)** (*n* = 3, mean ± SEM). **(F)** DNA quantification during mesoderm differentiation from day 7 to day 14, day 7 set to 1 (*n* = 5, mean ± SEM, *p < 0.05 vs. Ctrl, paired *t*-test + Bonferroni; ^#^*p* < 0.05 vs. day 7, ANOVA + Bonferroni). **(G)** Cell count on day 14 of differentiation relative to day 7 determined using the hemocytometer (*n* = 8). Medians are depicted as horizontal lines, boxes represent first and third quartiles and whiskers are maximal and minimal values. White circle indicates an outlier with a value between 1.5 and 3 times the interquartile range (IQR) (*p < 0.05 vs. Ctrl, Wilcoxon test + Bonferroni).

The initial mesoderm predifferentiation (Figure 1A) under standard control conditions was performed as described before (Diederichs et al., 2019). Upon 50–70% iPSC confluency, expansion medium was switched to mesodermal differentiation medium (DMEM high glucose w/o L-glutamine, 12.5% fetal calf serum, 2 mM L-glutamine, 1% NEAA, 0.1% β-mercaptoethanol [all from Gibco], 100 U/mL penicillin and 100 μg/mL streptomycin [Merck-Millipore, Darmstadt, Germany], supplemented with 4 ng/mL FGF-2 [Active Bioscience]). At day zero, 5 μM CHIR99021 (Tocris, Wiesbaden, Germany) was added and later removed with the medium exchange 24 h later. Where appropriate, 0.025% dimethyl sulfoxide (DMSO) was added as solvent control. The medium was exchanged daily. At days 3 and 4, CHIR-treated cells received mesodermal differentiation medium with or without the WNT inhibitor IWP2 (5 μM, Tocris) or 0.1% DMSO. At day 7, the cells were detached using 0.05% trypsin/0.02% EDTA (Merck-Millipore) and reseeded into 0.1% gelatin-coated culture flasks at a density of 20 000 cells/cm^2^ with mesodermal differentiation medium, supplemented with 10 μM Y27632 for 48 h. Until day 14, the medium was exchanged twice.

At day 14, cells were subjected to 3D pellet culture with 5 × 10^5^ cells per pellet and chondrogenic medium consisting of high-glucose DMEM (Gibco) supplemented with 0.1 μM dexamethasone, 0.17 mM ascorbic acid 2-phosphate, 5 μg/mL transferrin, 5 ng/mL sodium selenite, 2 mM sodium pyruvate, 0.35 mM proline, 1.25 mg/mL bovine serum albumin (all from Sigma-Aldrich), 5 μg/mL insulin (Lantus^®^, Sanofi-Aventis, Frankfurt, Germany), penicillin/streptomycin, and supplemented with 10 ng/mL recombinant human transforming growth factor beta 1 (TGF-β1, Miltenyi Biotec GmbH, Bergisch Gladbach, Germany) for up to 42 days. The medium was exchanged three times a week, whereby loose cells that did not contribute to the pellet formation were flushed away.

### Western Blotting

The cells were harvested on ice in PhosphoSafe^TM^ extraction reagent (Merck-Millipore) supplemented with 1 mM Pefabloc^®^ SC (Sigma-Aldrich). Protein concentration in the lysates was determined using the Bradford assay kit (Sigma-Aldrich). Proteins were separated via SDS-PAGE and blotted onto a nitrocellulose membrane (GE Healthcare, Munich, Germany). The membrane was cut horizontally and immunostaining was performed using the following antibodies: anti-total β-catenin (1:2000, Clone 14, BD Biosciences, San Jose, CA, United States), anti-active β-catenin (1:2000, clone 8E7, Merck-Millipore) on the upper part of the membrane and anti β-actin (1:10000, clone AC-15, Genetex, Irvine, CA, United States) on the lower part. Bands were visualized with peroxidase-coupled secondary antibodies using enhanced luminol-based chemiluminescent detection. After probing for total β-catenin, the upper membrane part was treated for 15 min with Restore^TM^ Plus Western Blot Stripping Solution (Thermo Fisher Scientific, Schwerte, Germany) and removal of the signal was confirmed, before re-probing for active β-catenin.

### DNA Quantification

Pelleted cells or chondrogenic micromass pellets were digested using 200 μg/mL proteinase K (Thermo Fisher Scientific) in 0.05 M Tris (Merck-Millipore) and 1 mM calcium chloride (Sigma-Aldrich) over night at 60°C. The DNA content was determined using the Quant-iT PicoGreen double strand DNA kit (Invitrogen Life Technologies) according to the manufacturer’s instructions and determined by fluorescence measurement at 485/535 nm. Standards were prepared from λ-DNA.

### Quantitative Gene Expression Analysis

Total RNA was isolated by a standard guanidinium thiocyanate/phenol extraction procedure using peqGOLD TriFast (Peqlab, Erlangen, Germany) according to the manufacturer’s instructions. cDNA was synthesized from 500 ng of total RNA using Omniscript^®^ reverse transcriptase (Qiagen, Hilden, Germany) according to the manufacturer’s protocol. Relative gene expression levels were determined by quantitative PCR (qPCR) analysis using SybrGreen (Thermo Fisher Scientific) and the LightCycler^®^ 96 system (Roche Diagnostics) with the gene-specific primers shown in Supplementary Table 1. Specificity of the PCR products was confirmed by melting curve analysis and agarose gel electrophoresis. Gene expression was normalized to the Ct value of the reference gene *RPL13* and relative gene expression was calculated as 1.8^–Δ^^Ct^.

### Flow Cytometry

Cells were fixed and labeled with anti-PDGFRα (1:25, clone aR1, overnight at 4°C, BD Biosciences) phycoerythrin-conjugated antibody. Unstained cells were used as controls to adjust the gates. The surface marker-positive cells were quantified with a MACSQuant Flow Cytometer and the results were analyzed using MACSQuantify Version 2.11.1746.19438 (both Miltenyi Biotec).

### Histology

Pellets were fixed in 4% formaldehyde for 2 h, dehydrated and paraffin-embedded. Five-micrometer-sections were deparaffinized, rehydrated and stained with Safranin O (0.2% in 1% acetic acid) and counterstained with fast green (0.04% in 0.2% acetic acid) according to standard histological protocols. Immunohistological staining against collagen type II was performed as described previously (Winter et al., 2003). Sections were treated with 4 mg/mL hyaluronidase followed by 1 mg/mL pronase (Roche), and unspecific binding sites were blocked with 5% bovine serum albumin. Collagen II was detected with a 1:1000-diluted monoclonal mouse anti-human collagen type II antibody (clone II-4C11, overnight at 4°C, MP Biomedicals) and visualized with biotinylated goat anti-mouse secondary antibody (1:500; 30 min, at room temperature; Dianova, Hamburg, Germany), streptavidin-alkaline phosphatase and fast red (Vector Laboratories, Burlingame, CA, United States).

To determine the pellet volume via histomorphometry, safranin O images were analyzed using the ImageJ Software (National Institute of Health, Bethesda, MD, United States). The horizontal and vertical diameters of the pellets were measured using the measure function with the scale set according to the scale bar. The radius was calculated from the mean diameter. The approximate volume was calculated using the sphere volume formula $V=\left(\frac{4}{3}\right)\times \pi \times r^{3}$.

### Whole Transcriptome cDNA Microarray

Confluent cells were harvested at day 14 of mesoderm differentiation. RNA was isolated as described above. Quality control of total RNA, labeling, array hybridization, and microarray scanning were performed at the German Cancer Research Center Genomics Core Facility (Heidelberg, Germany) using human Clariom^TM^ S Assay (Thermo Fisher Scientific). Data were extracted for all individual probes of a set and outliers with a more than 2.5-fold difference from the mean were removed. Fluorescence signals were quantile-normalized.

### Bioinformatic Analysis

Using the quantile-normalized data, fold-differences between CHIR and control cells were calculated. Statistical overrepresentation was tested for the PANTHER Gene Ontology-Slim Cellular Component category using the PANTHER software (^1^ Version 14.1, released 12 March 2019; Mi et al., 2019), submitting the list of all genes with a more than two-fold different expression value between the two groups.

### Aggregation and Condensation Assay

After mesodermal predifferentiation, day 14 progenitor cells were detached with trypsin/EDTA, washed with phosphate buffered saline and re-suspended in chondrogenic induction medium. 5 × 10^5^ cells per well were seeded into uncoated 24 well tissue culture plates with three biological replicates per group as previously described (Diederichs et al., 2016; Buchert et al., 2019). Aggregation and condensation were observed and photographed hourly during the first 6 h and daily thereafter. Aggregation was considered completed when cells had finished assembling into 3D structures. The experiment was ended in the control group at day 7 and in the CHIR group at day 14.

### Statistical Analysis

The number of independent experiments performed for each analysis is given in the figure captions. Data were analyzed using SPSS-25. For the microarray analysis, see separate section above. Means and standard error of the mean (SEM), as well as medians, first and third quartiles, outliers (value between 1.5 and 3-fold of the interquartile range, IQR) and extreme values (above three-fold IQR) were calculated. Where mentioned, fold changes were calculated, setting the appropriate control to 1. For time courses, differences within one group over time were assessed via one-way ANOVA and *post hoc* Bonferroni, and differences between groups at the same time point via paired *t*-test. In experiments analyzing single time points, differences were analyzed using Wilcoxon test. Bonferroni correction was applied to account for multiple testing.

## Results

### Short Initial CHIR Treatment Enhanced Proliferation During Mesodermal Predifferentiation

To mimic the short initial WNT/β-catenin pulse at the onset of embryonic mesoderm development, cells from two independent iPSC lines, IMR and CB, were treated with 5 μM of the WNT activator CHIR99021 (CHIR) at initiation of differentiation (day 0, Figure 1A). According to Western blotting, already the switch from expansion medium to the serum-containing mesodermal predifferentiation medium upregulated active β-catenin levels in DMSO controls (Figure 1B). However, CHIR treatment significantly raised mean active β-catenin levels 5.5-fold compared to DMSO controls according to densitometric evaluation (*n* = 5 experiments) with β-actin used as reference (Figure 1C). To subsequently terminate the induced WNT signal, CHIR was removed from the medium after 24 h. Consequently, active β-catenin levels declined and reached control levels at day 3. Similar results were also obtained with the CBiPSC line (Supplementary Figure 1A). Beyond removal of the activator in one group, a further group obtained 5 μM of the WNT inhibitor IWP2 on days 3 to 5 to actively inhibit WNT/β-catenin. However, this did not further decrease active β-catenin levels below the CHIR group at days 5 and 7 (Figures 1D,E). Thus, removal of CHIR after 24 h was sufficient and active suppression using IWP2 was not needed for silencing of WNT/β-catenin activity. Altogether, CHIR-treatment at the initiation of mesoderm differentiation of iPSCs recapitulated a WNT pulse known from the onset of embryonic mesoderm formation.

Continuously higher medium consumption of the CHIR group after removal of the activator led us address potential differences in cell numbers from day 7 onward. At this time point, the original cell colonies were fragmented into a single-cell suspension, which was re-seeded with a defined cell density, thus providing a reference for DNA quantification. CHIR-treated IMR and CBiPSC cultures had a significantly higher DNA content compared to controls from day 10 onward (IMR 5.09-fold on day 14, *p* < 0.05, CB 2-fold on day 14, *p* = 0.064, *p* = 0.051, Figure 1F and Supplementary Figure 1B), reaching a 5.06-fold (IMR) or 3.7-fold (CB) higher cell count at day 14 than in controls (Figure 1G and Supplementary Figure 1C). IWP2 treatment had no further benefit for cell proliferation (Figures 1F,G). Altogether, pulsing iPSCs with the WNT activator CHIR at initiation of mesoderm differentiation significantly increased cell proliferation and strongly enhanced cell yield during mesodermal predifferentiation, independent of an active WNT re-silencing step, which was, thus, discontinued in further experiments.

### Enhanced Silencing of Pluripotency Markers

**FIGURE 2:**
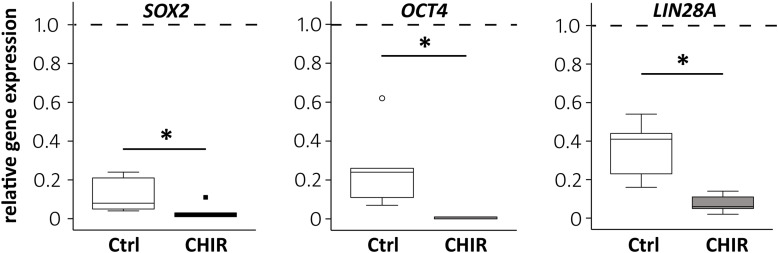
Regulation of the pluripotency-associated genes after CHIR treatment. Gene expression of *SOX2, OCT4*, and *LIN28A* at day 14 of IMR-iPSC mesodermal predifferentiation in CHIR-treated cells compared to control (Ctrl) was determined by qPCR and *RPL13* was used as reference. Relative gene expression compared to expression in iPSCs at d0 of differentiation (dashed line) is depicted. White circle indicates an outlier with a value between 1.5 and 3 times the IQR, black square indicates an extreme outlier above 3 times IQR (*n* = 5, *p < 0.05, Wilcoxon test).

To test the effects of the initial WNT/β-catenin pulse on cell development, the expression of pluripotency markers during mesodermal predifferentiation was analyzed by qPCR. SOX2 and OCT4 were already significantly repressed at termination of CHIR treatment compared to controls (Supplementary Figure 2A). At day 14, *SOX2, OCT4*, and *LIN28A* were all considerably downregulated compared to day 0 iPSCs in both groups (Figure 2), and importantly, this decrease was significantly stronger in the CHIR group than in controls (*OCT4* −56.1-fold, *SOX2* −3.7-fold, and *LIN28A* −5.2-fold). This demonstrated that the initial WNT/β-catenin pulse, although transient, supported the permanent exit from pluripotency and suggested an advanced differentiation status compared to the control group.

### Short Initial CHIR Treatment Improved Mesoderm Formation

**FIGURE 3:**
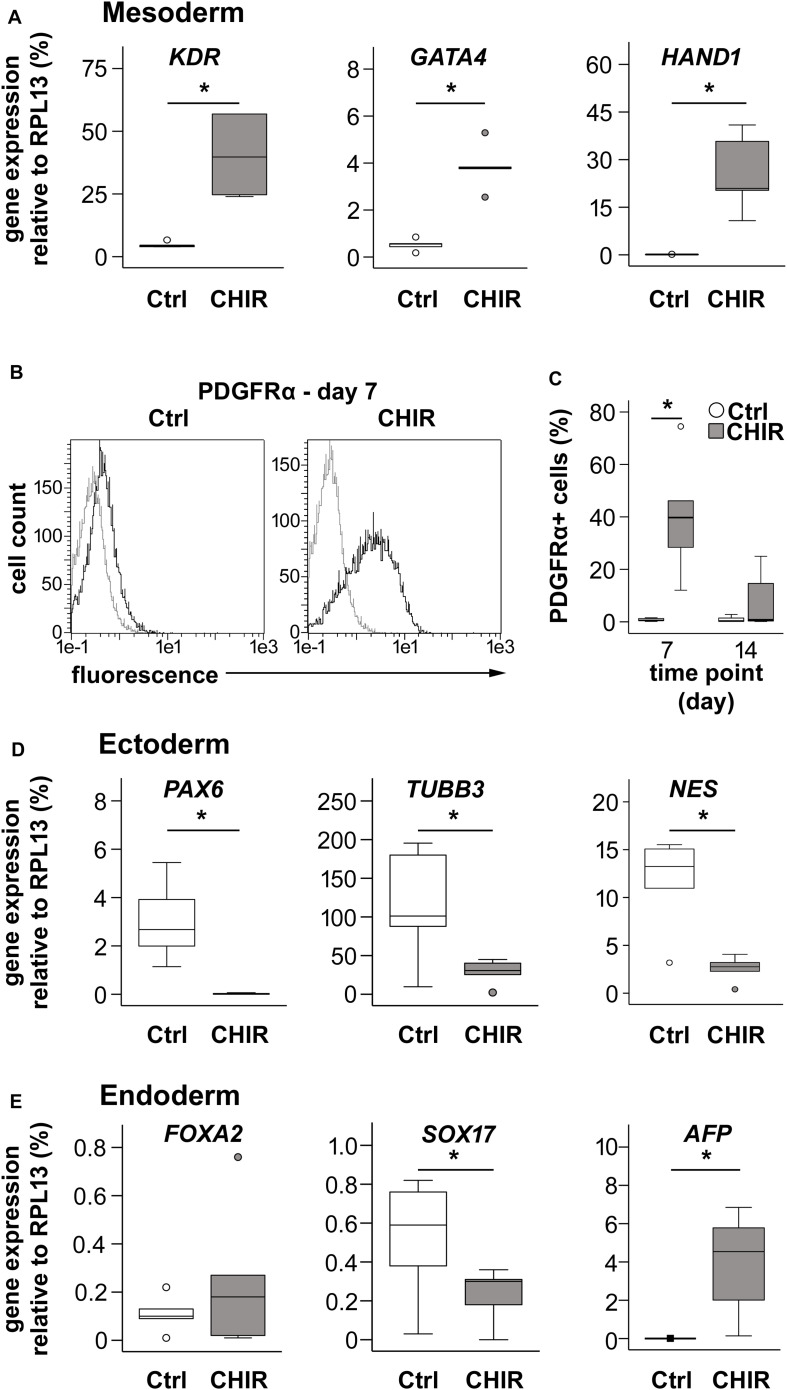
Effects of CHIR treatment on differentiation toward the three germ layers during IMR-iPSC mesoderm differentiation. Mesoderm differentiation was assessed by **(A)** gene expression on day 14 of the mesoderm markers *KDR, GATA4*, and *HAND1*, using qPCR and *RPL13* serving as reference (*n* = 5); and by **(B)** flow cytometric analysis of PDGFRα expression at day 7 of differentiation. Representative histograms depict immuno-labeled cells (black lines) compared to unstained control cells (light gray lines), which were used to adjust the gates. **(C)** Quantification of percentage of PDGFRα-positive cells at day 7 and day 14 in Ctrl and CHIR groups (*n* = 5). Gene expression of specified **(D)** ectoderm markers and **(E)** endoderm markers was assessed by qPCR at day 14 and *RPL13* served as reference gene. Circles in all boxplots depict outliers with values between 1.5 and 3 times the IQR, black square indicates an extreme outlier above 3 times IQR (*n* = 5, *p < 0.05, Wilcoxon test), Ctrl = control.

Next, we investigated whether enhanced exit from pluripotency in CHIR-treated cells was accompanied by increased expression of lineage-specific markers and whether the desired mesodermal differentiation was favored over ectodermal and endodermal specification. At day 1, the transient early mesoderm markers *T* and *MIXL1* were strongly increased in CHIR-treated IMR cells compared to controls along with the mesodermal markers *KDR* and *GATA4*, indicating improved mesoderm induction in the CHIR group (Supplementary Figure 2B). Importantly, expression of *KDR* and *GATA4* remained significantly elevated along with enhanced *HAND1* in the CHIR group at day 14 (8.6-, 240.6-, and 7.4-fold, respectively, Figure 3A). This indicated that the CHIR pulse rapidly induced a fate decision to commit cells to mesoderm development. To confirm this, we analyzed levels of the common mesoderm marker PDGFRα, which is transiently expressed during *in vitro* human pluripotent stem cell mesoderm differentiation with a peak during the first week of differentiation (Tan et al., 2013; Adkar et al., 2019). In line with increased mesoderm marker expression, short initial CHIR treatment significantly increased the mean number of PDGFRα-positive cells at day 7 over 50-fold in IMR cells and 12-fold in the CB line compared to controls (Figures 3B,C and Supplementary Figure 3). While, as expected, PDGFRα was downregulated until day 14, the difference between CHIR and controls was reduced (Figure 3C), but remained significant in the CB line (Supplementary Figure 3B). Importantly, the common ectodermal markers *PAX6, TUBB3*, and *NES* were significantly less expressed in the CHIR group than in controls (−158.1, −4.0, and −4.6-fold, respectively; Figure 3D), while for endodermal markers a less consistent response was observed. *FOXA2* remained unchanged by the CHIR pulse, but *SOX17* was significantly lower (2.2-fold), and *AFP* significantly higher expressed (1950-fold) in CHIR-treated cells than in the controls (Figure 3E). Overall, mRNA and protein analysis demonstrated that the early WNT/β-catenin pulse enhanced mesodermal differentiation of iPSCs and inhibited misdifferentiation into the ectodermal lineage.

### Enhanced Aggregation and Condensation

**FIGURE 4:**
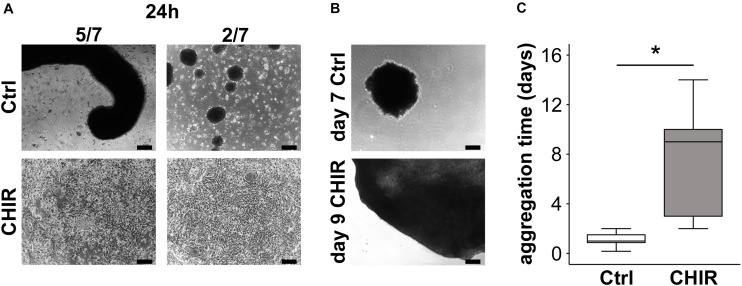
Effects of short initial CHIR treatment on aggregation and condensation of mesodermal progenitors. Condensation assay for control (Ctrl) and CHIR-treated IMR-iPSCs after **(A)** 24 h and **(B)** after cells completed aggregation. Representative experiments are shown, and numbers of typical experiments are indicated (scale bar = 200 μm). **(C)** Time until cells finished assembling into 3D structures (considered as completed aggregation) was determined microscopically (*n* = 7, *p < 0.05, Wilcoxon test).

In order to assess, whether the WNT-enhanced mesodermal differentiation would yield more aggregating mesodermal progenitors with a high capacity to condense, we seeded the iPSC-derived day 14 cells at high-density on non-coated cell culture dishes with chondrogenic medium. While control cells quickly formed multiple free-floating small aggregates and only few cells attached to the plastic surface (Figure 4A), the CHIR-treated cells aggregated into a plastic-adhering cell sheet, which subsequently condensed into one large pellet over 2 to 14 days (mean of 7 days; Figures 4B,C). Only a negligible number of free-floating cells was observed in the CHIR group, indicating that the vast majority of cells contributed to the cell sheet and, thus, to pellet formation. In contrast, only a minority of control cells assembled into one primary aggregate per well (5/7 experiments) or several small aggregates per well (2/7 experiments) within 48 h, without going through a plastic-adherence phase, while the vast number of cells remained free-floating (Figure 4A, top). These cells were then lost during routine medium exchanges (Figure 4B, top). Conclusively, the initial WNT/β-catenin pulse supported aggregation and plastic adherence of cells, thereby enhancing the number of mesodermal progenitors capable to form a cell sheet undergoing condensation and pellet formation.

### Increased Cell Survival and Tissue Yield During Chondrogenesis

**FIGURE 5:**
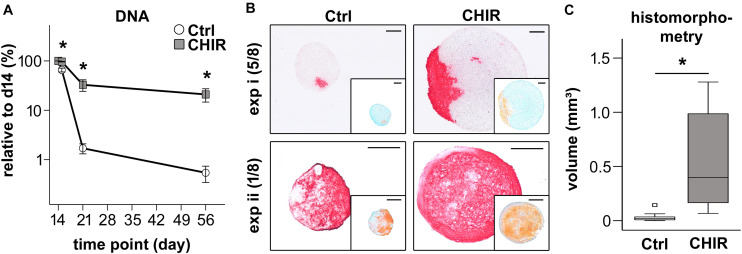
Chondrogenic differentiation of control (Ctrl) and CHIR-treated IMR-iPSCs. **(A)** DNA quantification at day 15, 21, and 56 of differentiation relative to day 14 (mean ± SEM, *n* = 3–10, ^∗^*p* < 0.05 vs. Ctrl, paired *t*-test). **(B)** Histological analysis after 42 days of chondrogenesis. Collagen type II deposition was examined by immunostaining and proteoglycan deposition by safranin O staining (insert). Two representative experiments (i and ii) shown and the numbers of typical experiments are indicated (*n* = 8, scale bar = 200 μM). **(C)** Tissue volume at day 56 calculated from histomorphometric data of cartilage pellets, white square indicates an extreme outlier above 3 times the IQR (*n* = 11, ^∗^*p* < 0.05, Wilcoxon test).

Next, we tested, whether improved cell aggregation would also be observed in classical 3D micromass culture in tubes and whether less non-aggregating cells would be lost during pellet formation. Therefore, we monitored the DNA content of cultures from the switch to pellet culture at day 14 onward (Figure 5A). After 24 h, 96.7 ± 1.8% of the starting population remained in the CHIR group, while only 65.6 ± 8.2% of the initial cells were retrieved in control pellets, indicating improved pellet formation by CHIR-treated cells. Importantly, flushing of pellets during the first medium exchange (day 16) did not visibly remove any cells in the CHIR group, while a large number of cells was washed out in controls. Thus, the CHIR group was dominated by aggregating cells, and removal of non-aggregating cells like in the control group became obsolete.

As a consequence of improved pellet formation, DNA amounts in the CHIR group remained significantly higher than in controls after 1 and 6 weeks of chondrogenic pellet culture (19-fold at day 21, 39-fold at day 56, Figure 5A). In 7/17 experiments, control pellets disintegrated over time, while the initial short CHIR treatment rescued this failure. Multiplied with the above observed 5-fold increased proliferation until day 14, the mean overall cell yield appeared 200-fold higher in the CHIR group compared to control after 56 days. Increased cell survival in CHIR pellets compared to controls was also evident in the CB line (Supplementary Figure 4A). In this iPSC line, all control pellets (*n* = 8 experiments) dissociated completely with time, which was rescued by the initial CHIR pulse.

In line with higher DNA amounts, histological assessment of pellets at day 56 of differentiation revealed a strongly increased pellet size in the CHIR group compared to controls (Figure 5B and Supplementary Figure 4B). Histomorphometric evaluation according to the idealized ellipsoid volume formula using the diameter of the central sections showed a 17.8-fold larger mean pellet volume for the CHIR group (Figure 5C). Interestingly, the collagen type II and proteoglycan positive area was larger in the CHIR group in the majority of experiments. However, compared to the total pellet size, the relative area of positive proteoglycan and collagen type II staining appeared similar in both groups (Figure 5B). In few cases (2/8), successful chondrogenesis was observed in controls but not in the CHIR group (data not shown). No additional benefits for pellet size as well as proteoglycan and collagen type II positive areas were observed in the IWP2 group described above (data not shown). Altogether, these experiments indicated that not all aggregating mesodermal progenitors developed into bona fide chondroprogenitors capable of cartilage formation and therefore some cultures may profit from further pathway modulations in future studies. Overall, this confirmed our hypothesis that short initial CHIR treatment could overcome the need of non-aggregating cell removal and thus improve cartilage tissue yield from iPSCs.

### Increased Extracellular Matrix and Adjusted Adhesion-Related Gene Expression

To gain more insights into the mechanisms and changes that allowed better aggregation and survival of the CHIR-treated mesodermal progenitors in chondrogenic culture, the global gene expression profile of day 14 mesodermal progenitors was compared. Whole transcriptome cDNA microarray analysis with IMR cells resulted in identification of 1365 genes with a more than two-fold change in gene expression after a CHIR pulse compared to the control. Of the 708 genes higher expressed in the CHIR group, 119 were more than five-fold elevated (top 10 in Table 1), while only 53 of the 657 genes with decreased expression surpassed the five-fold cut-off (top 10 in Table 2). This indicated a net gain in production for many gene products by short initial CHIR treatment.

**TABLE 1:** Mean microarray expression levels of top 10 higher expressed genes in CHIR-treated cells compared to standard control (Ctrl).

Gene symbol	Gene name	Ctrl	CHIR	Fold difference
***DCN***	**Decorin**	**56**	**4755**	**85.3**
*FRZB*	Frizzled-related protein	70	4578	65.5
***NPNT***	**Nephronectin**	**59**	**3364**	**56.7**
***LUM***	**Lumican**	**115**	**5517**	**47.9**
*ANXA8*	Annexin A8	58	2708	46.4
*RSPO2*	R-spondin 2	39	1425	36.1
*ANXA8L1; ANXA8*	Annexin A8-like 1; annexin A8	78	2382	30.6
*COBLL1*	Cordon-bleu WH2 repeat protein like 1	62	1783	28.8
*GUCY1A3*	Guanylate cyclase 1, soluble, alpha 3	45	1228	27.5
*AQP1*	Aquaporin 1 (Colton blood group)	128	3400	26.7

*Bold: genes confirmed by qPCR.*

**TABLE 2:** Mean microarray expression levels of top 10 lower expressed genes in CHIR-treated cells compared to standard control (Ctrl).

Gene symbol	Gene name	Ctrl	CHIR	Fold difference
*NT5E*	5-nucleotidase, ecto (CD73)	2287	86	−26.7
***SPP1***	**Secreted phosphoprotein 1**	**2318**	**90**	−**25.7**
*ANKRD1*	Ankyrin repeat domain 1 (cardiac muscle)	2801	120	−23.4
*GREM1*	Gremlin 1, DAN family BMP antagonist	1643	101	−16.2
***CDH6***	**Cadherin 6, type 2, K-cadherin (fetal kidney)**	**992**	**63**	−**15.7**
*F2RL1*	Coagulation factor II (thrombin) receptor-like 1	1339	114	−11.8
*NTM*	Neurotrimin	707	60	−11.7
*SERPINB7*	Serpin peptidase inhibitor, clade B (ovalbumin), member 7	532	47	−11.4
*SERPINE1*	Serpin peptidase inhibitor, clade E (nexin, plasminogen activator inhibitor type 1), member 1	1996	182	−11.0
*ADGRL4*	Adhesion G protein-coupled receptor L4	764	71	−10.8

*Bold: genes confirmed by qPCR.*

**FIGURE 6:**
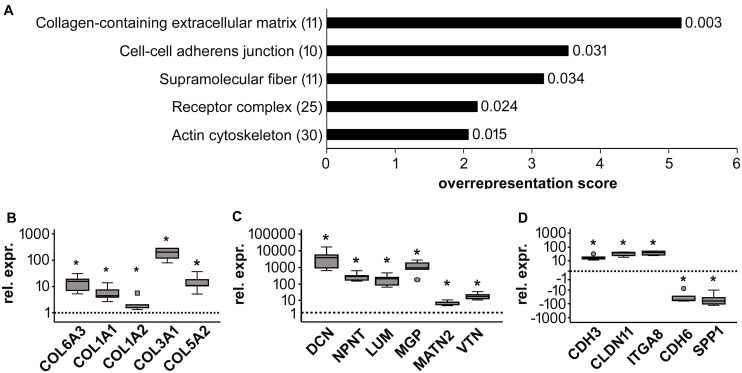
Global gene expression analysis of control (Ctrl) and CHIR-treated IMR-iPSC mesodermal progenitors (day 14). **(A)** PANTHER statistical overrepresentation analysis of all genes with a more than two-fold difference in mean expression levels between Ctrl and CHIR cells according to cDNA microarray data (*n* = 1). All significantly and more than two-fold overrepresented classes in the Gene Ontology-Slim Cellular Component category are depicted according to their overrepresentation score. Numbers in brackets indicate the number of genes included in each class (see also Tables 3–5) numbers behind each bar indicate the *p*-value. Differential regulation of designated collagen **(B)**, extracellular matrix **(C)** and adhesion-related **(D)** genes of interest was confirmed by qPCR analysis and *RPL13* served as reference gene. Relative gene expression (rel. expr.) compared to controls (dotted line) is depicted. Circles indicate outliers with a value between 1.5 and 3 times the IQR, the square indicates an extreme outlier above 3 times IQR (*n* = 5, *p < 0.05 vs. Ctrl, Wilcoxon test).

To classify major changes, all genes with a more than two-fold difference in mean expression levels between CHIR cells and controls were subjected to PANTHER analysis (Mi et al., 2019) and analyzed for statistical overrepresentation. Results were classified according to the main category ‘cellular component’ (PANTHER Gene Ontology-Slim) using two-fold overrepresentation as a cut-off. This classification yielded ‘collagen-containing extracellular matrix’ (ECM) as top hit (overrepresentation score 5.2) along with ‘supramolecular fiber’ as third hit (Figure 6A), indicating that ECM-related gene expression was one major WNT-induced change in mesodermal progenitors. Higher expression of 8/11 genes in the collagen-containing ECM category and 9/11 genes in the supramolecular fiber category in the CHIR group than in controls (Tables 3, 4) indicated a net gain in ECM-related gene expression. ECM-related genes identified by PANTHER contained all three main chains of collagen type VI (*COL6A1*, COL6A2, and COL6A3) as well as both collagen type I chains (COL1A1 and COL1A2). QPCR analysis based on five independent IMR experiments confirmed the significant higher expression of *COL6A3, COL1A1*, and *COL1A*2 (12-fold, 5-fold, and 2-fold, respectively; Figure 6B). Additionally, also *COL3A1* and *COL5A2*, which were also strongly elevated according to the array data (23.9-fold and 8.8-fold), were confirmed to be significantly higher expressed in CHIR than in control cells. Moreover, the non-collagenous molecules decorin, nephronectin and lumican, which were among the top 10 elevated transcripts (Table 1), as well as matrix GLA-protein (*MG*P, array: 25.4-fold up), matrilin 2 (*MATN2*, 8.1-fold up), and vitronectin (*VTN*, 6.7-fold up), which are all typically located in the ECM, were significantly increased after the CHIR pulse according to qPCR data from five independent experiments (Figure 6C). Overall, these data strongly indicated that the initial CHIR pulse enhanced the expression of ECM-related genes including several collagens in mesodermal progenitors at day 14.

**TABLE 3:** Mean microarray expression levels of the genes contained in the PANTHER collagen-containing extracellular matrix category shown in Figure 6A.

Gene symbol	Gene name	Ctrl	CHIR	Fold difference
***MATN2***	**Matrilin-2**	**110**	**890**	**8.1**
***COL6A3***	**Collagen, type VI, alpha 3**	**88**	**522**	**5.9**
*COL6A1*	Collagen, type VI, alpha 1	163	704	4.3
*COL6A2*	Collagen, type VI, alpha 2	144	585	4.1
***COL1A1***	**Collagen, type I, alpha 1**	**480**	**1591**	**3.3**
*COL14A1*	Collagen, type XIV, alpha 1	64	199	3.1
*COL5A1*	Collagen, type V, alpha 1	421	1153	2.7
***COL1A2***	**Collagen, type I, alpha 2**	**1871**	**4127**	**2.2**
*COL12A1*	Collagen, type XII, alpha 1	469	205	−2.3
*THBS2*	Thrombospondin-2	209	77	−2.7
*THBS1*	Thrombospondin-1	963	160	−6.0

*Bold: genes confirmed by qPCR.*

**TABLE 4:** Mean microarray expression levels of the genes contained in the PANTHER supramolecular fiber category shown in Figure 6A.

Gene symbol	Gene name	Ctrl	CHIR	Fold difference
***COL1A1***	**Collagen, type I, alpha 1**	**480**	**1591**	**3.3**
*SYNPO*	Synaptopodin	119	394	3.3
*TNNI1*	Troponin I type 1 (skeletal, slow)	65	199	3.1
*TNNC1*	Troponin C type 1 (slow)	137	397	2.9
*TNNC1*	Troponin I type 3 (cardiac)	137	397	2.9
*TNNT1*	Troponin T type 1 (skeletal, slow)	91	251	2.7
*COL5A1*	Collagen, type V, alpha 1	421	1153	2.7
***COL1A2***	**Collagen, type I, alpha 2**	**1871**	**4127**	**2.2**
*TNNT2*	Troponin T type 2 (cardiac)	115	254	2.2
*MYOZ2*	Myozenin 2	146	40	−3.7
*FHL2*	Four and a half LIM domains 2	419	103	−4.1

*Bold: genes confirmed by qPCR.*

The second hit of the Panther analysis was ‘cell-cell adherens junction,’ which was 3.5-fold overrepresented (Figure 6A). In line with the improved aggregation and condensation capacity of the CHIR-treated mesodermal progenitors, this suggested an adapted profile of adhesion molecule expression. Genes in this category were cadherins (6/10, Table 5), and qPCR analysis of partially independent IMR samples (*n* = 5) confirmed significant differential expression of the two most strongly regulated genes, *CDH3* (18-fold up) and *CDH6* (−25-fold down, Figure 6D). Also, the tight junction transcript *CLDN11* (array: 12-fold up) and the integrin encoding *ITGA8* (array: 8.8-fold up) were significantly higher expressed after the CHIR pulse according to qPCR data (*n* = 5). In addition, *SPP1*, which was the second most strongly decreased transcript according to array analysis (Table 2), was confirmed to be significantly lower expressed in the CHIR group compared to controls via qPCR (32-fold, *n* = 5, Figure 6D). This was in agreement with the reduced expression of *OCT4*, which is known to regulate *SPP1* in embryonic stem cells (Guo et al., 2002; Chen et al., 2006). Overall, we propose that enhanced ECM-related gene expression and adjusted expression of adhesion-related genes is an important mechanism allowing better cell aggregation and consequently higher survival during chondrogenesis.

**TABLE 5:** Mean microarray expression levels of the genes contained in the PANTHER cell-cell adherens junction category shown in Figure 6A.

Gene symbol	Gene name	Ctrl	CHIR	Fold difference
***CDH3***	**Cadherin 3, type 1, P-cadherin (placental)**	**90**	**515**	**5.7**
*CDH3*	Cadherin 15	90	515	5.7
*PKP2*	Plakophilin 2	630	3604	5.7
*LRRC1*	Leucine rich repeat containing 1	89	381	4.3
*CDH12*	Cadherin 12, type 2 (N-cadherin 2)	53	141	2.7
*CDH7*	Cadherin 7, type 2	77	186	2.4
*PVRL3*	Poliovirus receptor-related 3	500	243	−2.1
*PVRL1*	Poliovirus receptor-related 1 (herpesvirus entry mediator C)	202	77	−2.6
*CDH13*	Cadherin 13	419	110	−3.8
***CDH6***	**Cadherin 6, type 2, K-cadherin (fetal kidney)**	**992**	**63**	−**15.7**

*Bold: genes confirmed by qPCR.*

## Discussion

The inherent ability to non-invasively give rise to any adult tissue including cartilage, bone, skeletal and heart muscle in theoretically unlimited amounts renders iPSCs highly attractive for regenerative medicine, *in vitro* disease modeling, and pharmacological screens. However, the necessity to select specific cell subsets from heterogenically differentiated mesodermal populations to facilitate subsequent specification into chondrocytes, muscle cells or cardiomyocytes makes iPSC *in vitro* differentiation currently extremely inefficient. To overcome this hurdle, more knowledge on major signaling pathways controlling mesodermal differentiation of pluripotent stem cells *in vitro* is needed. In this study, we showed that a short initial WNT/β-catenin activation pulse enhanced not only mesoderm formation and exit from pluripotency, but also reduced ectodermal misdifferentiation and improved cell proliferation. Importantly, our data indicated for the first time to our knowledge that the WNT/β-catenin pulse acted via enhancing expression of collagens and other ECM-related molecules and accordingly adjusting cell adhesion molecule-related gene expression. In line with our initial hypothesis, the WNT-induced mesodermal progenitors showed a strongly increased capability to aggregate and to undergo mesodermal condensation thereafter. Importantly, this made the removal of non-aggregating cells obsolete and, thus, overcame the need for selection of specific subsets of mesodermal cells. As hypothesized, better aggregation after the WNT pulse was strongly beneficial for subsequent chondrogenesis, since now the majority of cells participated in pellet formation and consequently, the overall tissue yield drastically increased.

An important novelty of our study is to follow up and unravel the effects of the initial WNT pulse throughout the entire differentiation of iPSCs via mesodermal progenitors into chondrocytes and to decipher which cell functions were governed by WNT activation. The immediate WNT effect was to induce better mesoderm formation, while concurrently enhancing the exit from pluripotency and suppressing ectodermal misdifferentiation. This is in agreement with the well-described WNT-induced mesoderm formation and previous studies showing that WNT/β-catenin signaling induced the expression of primitive streak and early mesoderm markers, such as KDR and PDGFRα in human and mouse ESCs and iPSCs *in vitro* (Ueno et al., 2007; Wang and Nakayama, 2009; Wang et al., 2010; Tan et al., 2013; Shelton et al., 2014; Funa et al., 2015). Moreover, our observations are in line with human ESC cultures that were reported to downregulate *OCT3/4* and *NANOG* transcription as well as expression of the ectoderm markers *PAX6* and *TUBB3* upon WNT treatment during expansion (Davidson et al., 2012). Other groups previously utilized WNT activation for the mesodermal differentiation of human iPSCs even for later specification into chondrocytes, however, mostly in combination with additional pathway manipulations and without following up the specific WNT effects (Umeda et al., 2012; Yamashita et al., 2015; Adkar et al., 2019). Investigations 2 weeks after the WNT pulse identified here for the first time to our knowledge that WNT signaling acted via stimulation of ECM expression and altered cell-adhesion-related gene expression during mesodermal iPSC differentiation, thereby boosting the amounts of aggregating mesodermal progenitors and, thus, made cell selection obsolete. Of note, we demonstrated here also that a short initial WNT/β-catenin pulse was sufficient to induce and maintain strong effects over weeks of differentiation, even though active β-catenin levels were back to control niveau already at day 3. This indicated that WNT activation induced a fate decision (commitment to mesoderm development), after which external WNT signals appeared dispensable. Interestingly, continuous CHIR treatment of ESC cultures was previously shown to specify the induced primitive streak phenotype via mesendoderm into an endodermal fate (Tan et al., 2013). Derivation of PDGFRα and KDR-positive mesoderm was only possible, when CHIR treatment was discontinued after maximally 2 days, as this was done in the current study.

For specification of cardiomyocytes from WNT-induced mesodermal progenitors an active WNT inhibition is needed to restrict the expression of the anti-cardial regulators MSX1 and CDX2 (Lian et al., 2012; Rao et al., 2016). In contrast, we observed here no beneficial effects of the WNT inhibitor IWP2 on cell survival, tissue yield and cartilage formation (results of chondrogenesis not shown). This was in line with the essential functions of *CDX2* in paraxial mesoderm and somite formation in transgenic mice and human ESCs (Chawengsaksophak et al., 2004; Mendjan et al., 2014) and the importance of *Msx1* for mesoderm and cartilage formation in the limb bud (Watanabe and Le Douarin, 1996).

In addition to mesoderm commitment, the WNT/β-catenin pulse also increased cell proliferation during the mesodermal predifferentiation. This was in line with a large body of literature supporting pro-proliferative and pro-survival activity of WNT signaling in various settings. For example, in limb bud organ cultures ectodermal Wnt signals promoted proliferation in the underlying mesoderm (ten Berge et al., 2008). Moreover, Wnt treatment or β-catenin overexpression enhanced proliferation of hematopoietic stem cells (Reya et al., 2003; Willert et al., 2003). In turn, disruption of WNT/β-catenin signaling halted proliferation of cardiomyocytes in the developing heart (Heallen et al., 2011), and of tumor cells (van de Wetering et al., 2002). Thus, the here observed increased number of mesodermal progenitors was not only a result of enhanced mesodermal commitment, but also of elevated proliferation.

Interestingly, one consequence of the WNT-induced mesoderm commitment was an increased expression of collagens and other ECM-related genes as well as an adjusted cell-adhesion-related gene expression profile. Although this may appear surprising, since apart from a study in zebrafish demonstrating that WNT/β-catenin activity could induce *col12a1* expression, WNT signaling is scarcely known as ECM inducer (Wehner et al., 2017). However, ECM production and changes in cell adhesion are typical characteristics of mesodermal cells in the developing embryo. In line, a recent study that compared global gene expression profiles at various stages during myogenic ESC differentiation reported upregulation of genes related to ECM organization that began in a phase representing the limb bud stage (Shelton et al., 2019). Thus, the observed changes in gene expression were in accordance with a continuously enhanced mesoderm specification. In line, the here described WNT-regulated ECM and cell adhesion-related genes were all previously described in connection with the acquisition or loss of the mesodermal phenotype. For example, collagen type I is typically upregulated upon fibrotic changes of hepatocytes and renal epithelial cells, during which these cells lose their original epithelial phenotype to become matrix-adherent mesenchymal cells (Rastaldi et al., 2002; Kaimori et al., 2010). The here observed elevated expression of collagen types III and V as well as matrilin 2 along with collagen I was in line with a frequent co-expression of these molecules during embryonic development and their formation of heterotypic fibrils (von der Mark and von der Mark, 1977; Keene et al., 1987; Niederreither et al., 1995; Birk, 2001; Piecha et al., 2002). Moreover, *COL6A3*, *DCN*, and *LUM*, which were upregulated after the WNT pulse here, are all typical markers for the switch to a mesenchymal phenotype in lens epithelial and tumor cells (Saika et al., 2004; Chandler et al., 2007; Mathias et al., 2010; Clarke et al., 2017; Huang et al., 2018; Meng et al., 2018). Interestingly, decorin and also nephronectin, were identified as direct downstream targets of canonical Wnt signaling in developing teeth, hematopoietic progenitor cells or osteoblasts (Ichii et al., 2012; Ikehata et al., 2017; Xiong et al., 2019). Along with nephronectin, also its ligand *ITGA8* was elevated upon CHIR-treatment (Brandenberger et al., 2001).

The most strongly elevated cadherin after the WNT/β-catenin pulse, CDH3, was reported to correlate with the loss of an epithelial phenotype of tumor cells upon gaining the ability to migrate and invade other tissues (Baek et al., 2010; Li et al., 2014), while CHD3 suppression apparently reversed this phenotype (Timmermans-Sprang et al., 2019). Also claudin-11 was described to be characteristic for mesodermal cells during embryo development (Bronstein et al., 2000) and tumor cell migration (Li et al., 2019). Knock-down of *Cdh6* induced a premature transition into the mesenchymal phenotype in the neural crest of chick embryos (Coles et al., 2007) in line with the here observed decrease of *CDH6* expression after the initial WNT pulse. Collectively, the here observed WNT-induced ECM and cell adhesion signature is characteristic for an advanced mesodermal phenotype, which only few cells developed under control conditions.

Importantly, a key cell function governed by early WNT induction in all analyzed experiments was the ability to interact with matrix, which according to our aggregation and condensation assay enabled the mesodermal progenitors to aggregate into a cell sheet and to undergo condensation. Importantly, this for the first time demonstrates that an initial WNT/β-catenin pulse strongly increased cell-matrix interaction and aggregation of iPSC-derived mesodermal progenitors and facilitated their condensation. In line with our data, Shelton et al. described that CHIR-induced ESC-derived mesodermal progenitors formed 3D cell clusters at day 8 and subsequently the specified myocytes and fused myotubes radiated outward from these 3D clusters (Shelton et al., 2014).

Notably, ECM is commonly known to be decisive for cell condensation. Already in 1970 Hornbruch and Wolpert discussed that secretion of ECM was very likely responsible for the cell movements during precartilage condensation (Hornbruch and Wolpert, 1970). Since then, it has been established that the local cell rearrangements in this process are mediated by ECM-driven movements rather than cell migration or localized proliferation (Toole et al., 1972; Frenz et al., 1989; Daniels and Solursh, 1991; Hall and Miyake, 1995). In a recent study, we demonstrated that poorly condensing iPSC-derived mesodermal cells were characterized by a lack of ECM-related gene expression including *COL6A3* and *DCN* (Buchert et al., 2019). Moreover, segregation into specific subpopulations confirmed lower *COL6A3* and *DCN* expression in the non-aggregating than in the aggregating cell fraction, suggesting that shortage of ECM-related gene expression was causal for low aggregation capacity. Remarkably, in the current study both, ECM-related gene expression and cell aggregation were rescued concomitantly by CHIR treatment, which further supports the importance of ECM components like collagen VI and decorin for the ability of mesodermal progenitors to aggregate.

We here demonstrated for the first time, that improved cell condensation subsequently rescued the high cell loss and low tissue yield during chondrogenesis. Whether this is also equally important for specification into other mesodermal downstream lineages, is an interesting question. Because WNT treatment also improved specification of mesodermal progenitors into the cardiac and myogenic lineage (Lian et al., 2012; Shelton et al., 2014), we postulate that high aggregation capacity is an important functional quality of mesodermal progenitors in general.

Of note, once committed to the mesoderm by WNT activation, further specification into matrix-interacting, aggregating progenitors with high expression of ECM-related genes and an adapted cell adhesion-related expression profile occurred spontaneously in our study in the FGF-2 and serum-containing mesodermal differentiation medium. In controls, where β-catenin activation remained low, only very few cells developed this desired phenotype in accordance with a lack of mesodermal commitment. This underscores the importance of proper initial mesoderm commitment for the success of downstream differentiation into chondrocytes or myocytes.

While overall the cartilage yield was increased, heterogeneity was observed at the stage of specification into chondroprogenitors. In some cases, the initial WNT/β-catenin pulse may have directed cells into a myogenic cell fate in line with increased expression of several troponins according to our array analysis (Table 4). Thus, after having solved the first important step of driving the majority of the cells into precartilage condensation by initial WNT activation, further studies are needed to clarify whether these aggregating cells are preferentially committed to the chondrogenic compared to the myogenic lineage and to identify the specific stimuli that drive downstream commitment into chondroprogenitors. Several previous studies reported that modulation of the activin A, bone morphogenetic protein, TGF-β, FGF, retinoic acid, hedgehog, insulin-like growth factor, and platelet-derived growth factor pathways in highly differing combinations and various temporal sequences were beneficial for deriving chondrocytes from human iPSCs (Umeda et al., 2012; Wu et al., 2013; Craft et al., 2015; Yamashita et al., 2015; Adkar et al., 2019). However, none of these studies has been successfully reproduced independently and all still rely on cell selection procedures. This clearly demonstrates that our knowledge on the necessary pathways and their functions is still incomplete. Thus, functional implications of more individual pathways should be deciphered in the future to better guide the iPSC-derived mesodermal progenitors into the desired mature phenotype.

### Conclusion

A short WNT/β-catenin activation pulse at initiation of mesodermal iPSC differentiation was a key step to enhance mesoderm commitment and exit from pluripotency, to reduce ectodermal misdifferentiation and increase cell proliferation. Acting via stimulation of ECM expression and strongly improved cell aggregation, WNT activation made mesodermal progenitor selection before chondrogenesis obsolete. During subsequent chondrogenesis, enhanced aggregation was a key ability to allow the vast majority of the generated mesodermal progenitors to participate in pellet formation. Overall, we here identified extracellular matrix expression and cell aggregation as fundamental cell functions of mesodermal progenitors that depended on a WNT-induced mesoderm commitment. These mechanistic revelations will strongly advance not only chondrogenic, but also myogenic and cardiac differentiation of human iPSCs in the near future and bring these cells closer to application in regenerative medicine, disease modeling and drug screening.

## Data Availability Statement

The cDNA microarray data described in this manuscript can be found on: https://www.ebi.ac.uk/arrayexpress/, E-MTAB-9226.

## Author Contributions

UK contributed to conception and design, collection of data, data analysis and interpretation, visualization, manuscript writing, final approval of manuscript. JB contributed in data analysis and interpretation, manuscript writing, final approval of manuscript. AH contributed to provision of study material, final approval of manuscript. WR contributed to conception and design, financial support, administrative support, data interpretation, manuscript review and editing, final approval of manuscript. SD contributed to conception and design, financial support, administrative support, data analysis and interpretation, manuscript writing, final approval of manuscript. All authors approved the submitted version.

## Conflict of Interest

The authors declare that the research was conducted in the absence of any commercial or financial relationships that could be construed as a potential conflict of interest.

## Footnotes

1 http://pantherdb.org

## Supplementary Material

Supplementary Figure 1 Effects of short initial CHIR treatment on β-catenin regulation and proliferation during CBiPSC mesoderm differentiation. **(A)** Western blot analysis for active and total β-catenin during the first 7 days of mesoderm differentiation in CHIR-treated cells and the respective DMSO control in two independent experiments. **(B)** DNA quantification during mesodermal predifferentiation, day 7 set to 1 (*n* = 5, mean ± SEM). **(C)** Cell count on day 14 of differentiation relative to day 7 (*n* = 6, ^∗^*p* < 0.05, Wilcoxon test).

Supplementary Figure 2 Immediate effects of short initial CHIR treatment on expression of pluripotency and mesoderm markers in IMR-iPSCs. Gene expression of the specified **(A)** pluripotency **(B)** early mesoderm and **(C)** mesoderm markers was assessed by qPCR at day 1 of mesodermal differentiation. *RPL13* served as reference (*n* = 5, ^∗^*p* < 0.05, Wilcoxon-test). Circles indicate outliers with a value between 1.5 and 3 times the IQR, squares indicate extreme values above 3 times IQR.

Supplementary Figure 3 Effects of short initial CHIR treatment on CBiPSC mesoderm differentiation at day 7 and 14. **(A)** Immuno-labeled cells were subjected to flow cytometry for PDGFRα (black lines) and unstained control cells were used to adjust the gates (gray lines). **(B)** Quantification of PDGFRα-positive cells at day 7 and day 14 in control (Ctrl) and CHIR-treated cells. Black squares indicate extreme outliers above 3 times the interquartile range (*n* = 5, ^∗^*p* < 0.05, Wilcoxon test).

Supplementary Figure 4 DNA and histological analysis of chondrogenic 3D micromass pellets in control (Ctrl) and CHIR-treated CBiPSCs. **(A)** DNA quantification at day 21 and 56 of differentiation relative to day 14 (mean ± SEM, *n* = 3–6). **(B)** Proteoglycan deposition as assessed by safranin O staining after 56 days of differentiation (representative image of Ctrl and CHIR pellets, *n* = 8, scale bar = 100 μM).

Supplementary Table 1 Forward and reverse primers used for qPCR.
