# Supplementary figures and images for: Initial WNT/β-Catenin Activation Enhanced Mesoderm Commitment, Extracellular Matrix Expression, Cell Aggregation and Cartilage Tissue Yield From Induced Pluripotent Stem Cells
Source: Front Cell Dev Biol. 2020 Oct 30;8:581331. doi: 10.3389/fcell.2020.581331 (PMC7661475; doi:10.3389/fcell.2020.581331)

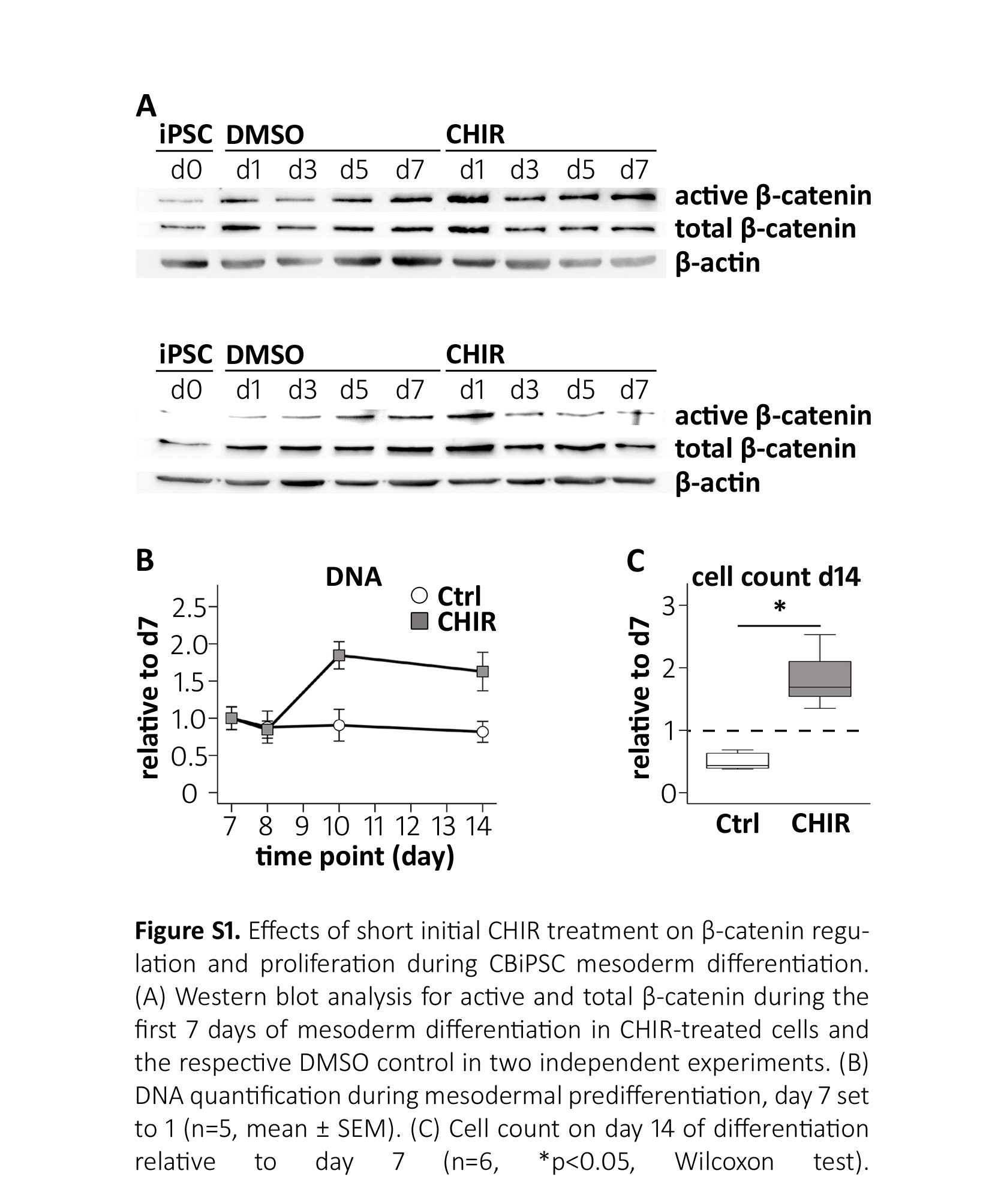

Supplement: Supplementary Figure 1 — Effects of short initial CHIR treatment on β-catenin regulation and proliferation during CBiPSC mesoderm differentiation. (A) Western blot analysis for active and total β-catenin during the first 7 days of mesoderm differentiation in CHIR-treated cells and the respective DMSO control in two independent experiments. (B) DNA quantification during mesodermal predifferentiation, day 7 set to 1 (n = 5, mean ± SEM). (C) Cell count on day 14 of differentiation relative to day 7 (n = 6, ∗p < 0.05, Wilcoxon test). [file Image_1.TIF]

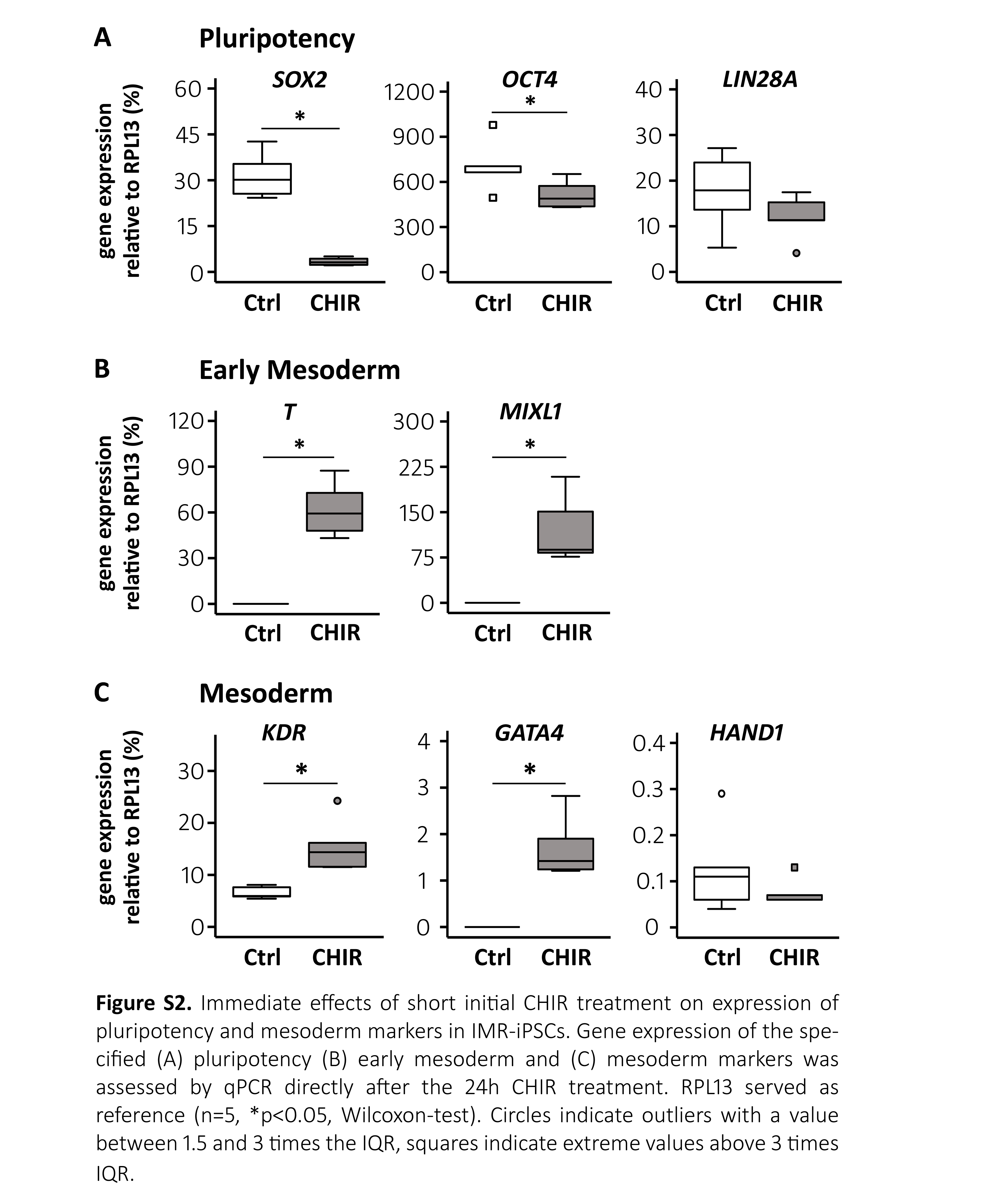

Supplement: Supplementary Figure 2 — Immediate effects of short initial CHIR treatment on expression of pluripotency and mesoderm markers in IMR-iPSCs. Gene expression of the specified (A) pluripotency (B) early mesoderm and (C) mesoderm markers was assessed by qPCR at day 1 of mesodermal differentiation. RPL13 served as reference (n = 5, ∗p < 0.05, Wilcoxon-test). Circles indicate outliers with a value between 1.5 and 3 times the IQR, squares indicate extreme values above 3 times IQR. [file Image_2.TIF]

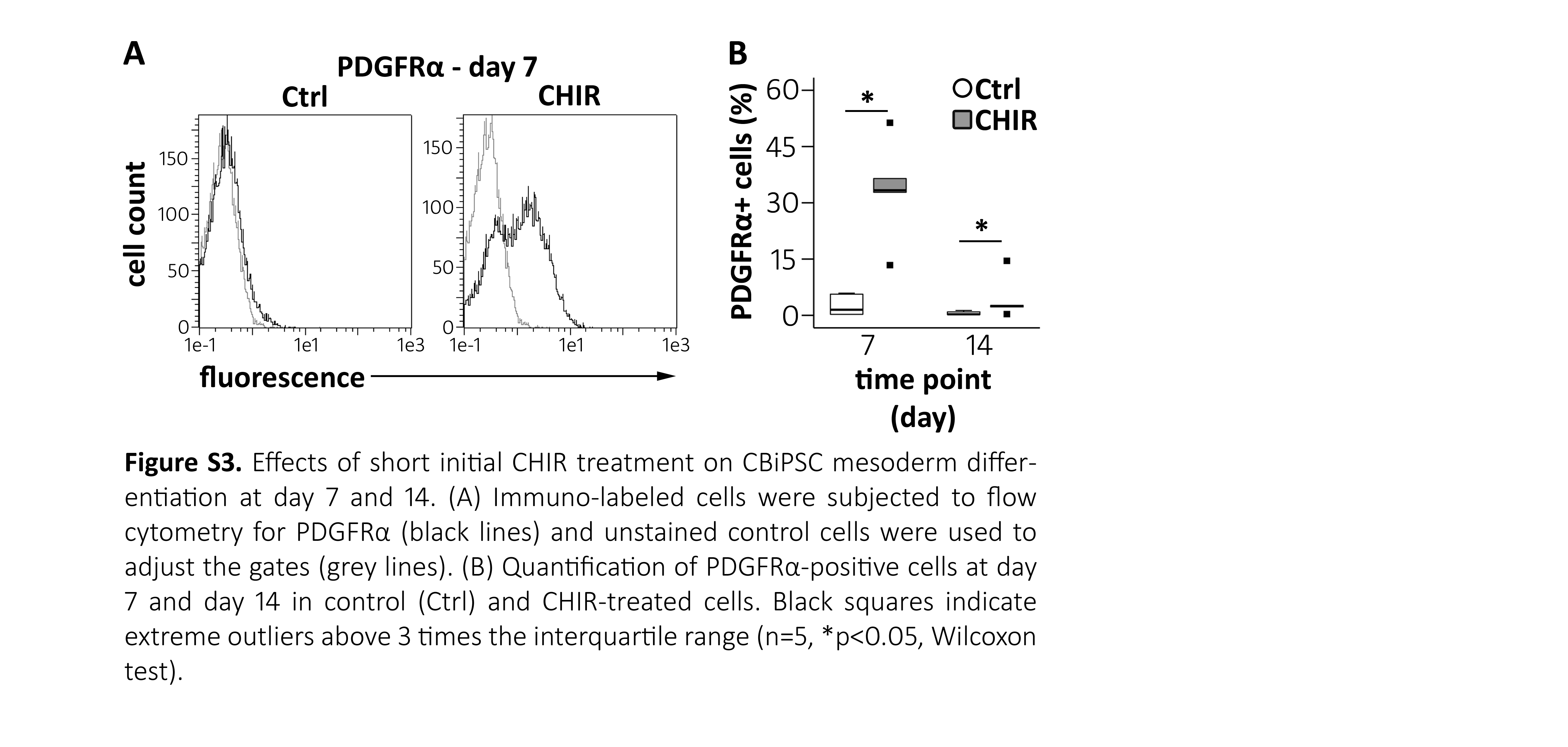

Supplement: Supplementary Figure 3 — Effects of short initial CHIR treatment on CBiPSC mesoderm differentiation at day 7 and 14. (A) Immuno-labeled cells were subjected to flow cytometry for PDGFRα (black lines) and unstained control cells were used to adjust the gates (gray lines). (B) Quantification of PDGFRα-positive cells at day 7 and day 14 in control (Ctrl) and CHIR-treated cells. Black squares indicate extreme outliers above 3 times the interquartile range (n = 5, ∗p < 0.05, Wilcoxon test). [file Image_3.TIF]

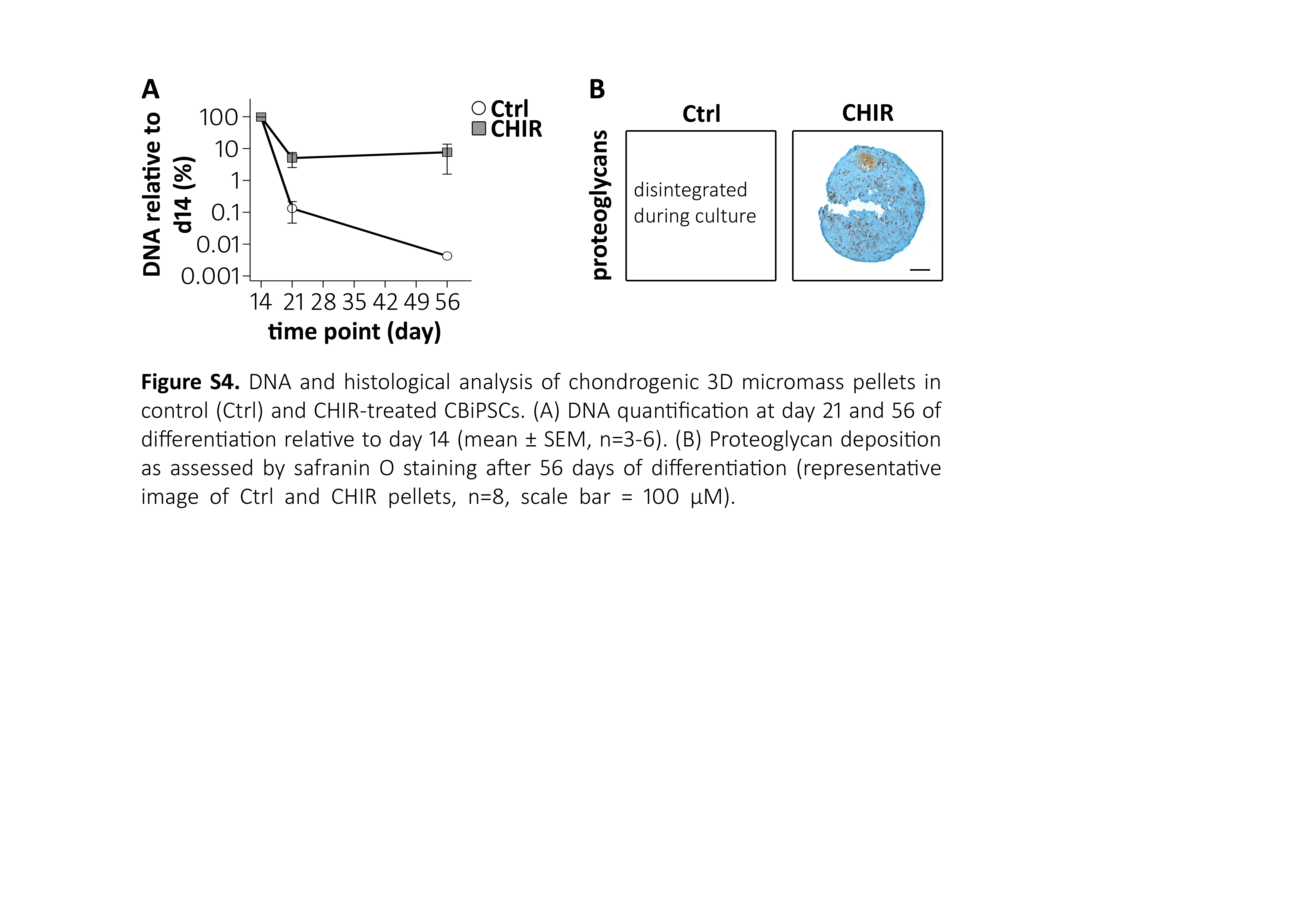

Supplement: Supplementary Figure 4 — DNA and histological analysis of chondrogenic 3D micromass pellets in control (Ctrl) and CHIR-treated CBiPSCs. (A) DNA quantification at day 21 and 56 of differentiation relative to day 14 (mean ± SEM, n = 3–6). (B) Proteoglycan deposition as assessed by safranin O staining after 56 days of differentiation (representative image of Ctrl and CHIR pellets, n = 8, scale bar = 100 μM). [file Image_4.TIF]
