# Supplementary material for: Initial WNT/β-Catenin Activation Enhanced Mesoderm Commitment, Extracellular Matrix Expression, Cell Aggregation and Cartilage Tissue Yield From Induced Pluripotent Stem Cells
Source: Front Cell Dev Biol. 2020 Oct 30;8:581331. doi: 10.3389/fcell.2020.581331 (PMC7661475; doi:10.3389/fcell.2020.581331)
Supplement: Supplementary Table 1 — Forward and reverse primers used for qPCR. [file Data_Sheet_1.PDF]

Table S1: Forward and reverse primers used for qPCR

Gene	Accession Number	Primer forward	Primer reverse
AFP	NM_001134.2	5'-CTTTGGGCTGCTCGCTATGA-3'	5'-GCATGTTGATTTAACAAGCTGCT-3'
CDH3	NM_001317196.2	5'-GCTACCGCATCCTGAGAGAC-3'	5'-ACCTCTGCCGTCCAGTAGAT-3'
CDH6	NM_001362435.2	5'-AGATGCTCAGATAAACACCACA-3'	5'-TCTGTCCATATCTGTGTGTCGA-3'
CLDN11	NM_005602.6	5'-CTGCTGCTGACTGTTCTTCC-3'	5'-CAACAAGGGCGCAGAGAG-3'
COL1A1	NM_000088.4	5'-GATGCCAATGTGGTTCGTGA-3'	5'-TCA GCT GGA TGG CCA CAT C-3'
COL1A2	NM_000089.4	5'-GGACTATGAAGTTGATGCTACT-3'	5'-GTCACTCCTTCAACATTATATTC-3'
COL6A3	NM_004369.4	5'-CACAACAAAGCCTGTAACCACC-3'	5'-CTGTTATCTCAAACACCTGGAC-3'
COL3A1	NM_000090.4	5'-TCGAGGCAGTGATGGTCAAC-3'	5'-TCCTGGGATGCCATTTGGTC-3'
COL5A2	NM_000393.5	5'-CTCAGGGAATTGATGGAGAACC-3'	5'-CAGAGCCAGGCATTAGTCCT-3'
DCN	NM_001920.5	5'-TCCCAACTTAGCCAAATTATTC-3'	5'-ACACAACTCTGCTAGACCTG-3'
FOXA2	NM_021784.4	5'-GGAGCAGCTACTATGCAGAGC-3'	5'-CGTGTTCATGCCGTTCATCC-3'
GATA4	NM_002052.3	5'-CGACACCCCAATCTCGATATG-3'	5'-GTTGCACAGATAGTGACCCGT-3'
HAND1	NM_004821.2	5'-CCATGCTCCACGAACCCTTC-3'	5'-CCTGGCGTCAGGACCATAG-3'
ITGA8	NM_003638.3	5'-TGGAACCAAAGAACCTATCGAG-3'	5'-GGTCCTTTTCTGGTGTCGGT-3'
KDR	NM_002253.3	5'-AATCTCTTGCAAGCTAATGCT-3'	5'-CTCCATACAGGAAACAGGTG-3'
LIN28A	NM_024674.6	5'-TTCGGCTTCCTGTCCATGAC-3'	5'-AGACCCTTGGCTGACTTCTT-3'
LUM	NM_002345.3	5'-ATGCCACACCACAAGATCCC-3'	5'-ACCACCAATCAATGCCAGGA-3'
MATN2	NM_002380.5	5'-GTCTGCAGGTGCAAACAAGG-3'	5'-TTTCAGGCAGTGGCAGGAAT-3'
MGP	NM_001190839.3	5'-GCAAATACCTTCATATCCCCTCA-3'	5'-GTAGCGTTCGCAAAGTCTGT-3'
MIXL1	NM_031944.2	5′-GGTACCCCGACATCCACTT-3′	5′-GCCTGTTCTGGAACCATACCT-3′
NES	NM_006617.2	5'-CTGCGGGCTACTGAAAAGT-3'	5'-GGAAGTTGGGCTCAGGACT-3'
NPNT	NM_001184692.2	5'-TGCATGAACACTTACGGCAG-3'	5'-CTGACAGTTTGCCATGGAGC-3'
OCT4	NM_203289.5	5'-CTGAAGCAGAAGAGGATCAC-3'	5'-GACCACATCCTTCTCGAGCC-3'
PAX6	NM_000280.4	5'-AACGATAACATACCAAGCGTGT-3'	5'-GGTCTGCCCGTTCAACATC-3'
RPL13	NM_012423.4	5'-CATTTCTGGCAATTTCTACAG-3'	5'-CAGGCAACGCATGAGGAAT-3'
SOX17	NM_022454.4	5'-GAGCCAAGGGCGAGTCCCGTA-3'	5'-CCTTCCACGACTTGCCCAGCAT-3'
SOX2	NM_003106.3	5'-GTGGTTACCTCTTCCTCCCACTC-3'	5'-CCTCCAGTTCGCTGTCCGGC-3'
SPP1	NM_001040058.2	5'-GCTAAACCCTGACCCATCTC-3'	5'-ATAACTGTCCTTCCCACGGC-3'
T	NM_003181.3	5′-TGCTTCCCTGAGACCCAGTT-3′	5′-GATCACTTCTTTCCTTTGCATCAAG-3′
TUBB3	NM_006086.4	5'-ATGAGGGAGATCGTGCACAT-3'	5'-CCCCTGAGCGGACACTGT-3'
VTN	NM_000638.4	5'-GGGTCTACTTCTTCAAGGGGAA-3'	5'-AATGAACTGGGGCTGTCTGG-3'
